# Application of Optical Coherence Tomography Angiography in True and Pseudo-Optic Disc Swelling

**DOI:** 10.1155/2024/1164635

**Published:** 2024-09-30

**Authors:** Kaveh Abri Aghdam, Ali Aghajani, Amin Zand, Samira Chaibakhsh, Pasha Anvari, Fatemeh Zahra Ijadi, Khalil Ghasemi Falavarjani

**Affiliations:** ^1^ Eye Research Center Eye Department The Five Senses Health Institute Rassoul Akram Hospital School of Medicine Iran University of Medical Sciences, Tehran, Iran; ^2^ Isfahan Eye Research Center Department of Ophthalmology Isfahan University of Medical Sciences, Isfahan, Iran; ^3^ Clinical Research Development Unit Shafa Hospital Kerman University of Medical Sciences, Kerman, Iran; ^4^ Rajaie Cardiovascular Medical and Research Institute Iran University of Medical Sciences, Tehran, Iran; ^5^ Stem Cell and Regenerative Medicine Research Center Iran University of Medical Sciences, Tehran, Iran

## Abstract

**Purpose:**

We evaluated the optic disc microvasculature in healthy subjects and patients with optic nerve head drusen (ONHD), active papilledema, and acute nonarteritic anterior ischemic optic neuropathy (NAION) using optical coherence tomography angiography (OCTA).

**Methods:**

This prospective, comparative case series included sixteen eyes with ONHD, thirty-one eyes with active papilledema, sixteen eyes with acute NAION, and thirty-two healthy eyes. The Optovue AngioVue OCT and OCTA Imaging System recorded peripapillary retinal nerve fiber layer (RNFL) thickness and vessel density maps from the radial peripapillary capillary (RPC) slab.

**Results:**

Average RNFL thicknesses were greater in eyes with ONHD, papilledema, and NAION compared to control eyes (all *Ps* < 0.001), but this parameter did not differ among patient groups. The mean peripapillary vessel density did not differ between the ONHD and control groups (*P*=1.000), nor between the NAION and papilledema groups (*P*=0.216). However, this value in the ONHD and control groups was significantly higher than in the NAION and papilledema groups (all *Ps* < 0.05).

**Conclusion:**

RPC density is influenced during the progression of conditions such as ONHD, papilledema, and NAION. Although a decrease in vessel density values has been observed in cases of true disc edema, further research is necessary to assess the potential of OCTA in differentiating between true and pseudo-optic disc edema.

## 1. Introduction

Optic disc swelling can occur in different conditions, including papilledema and nonarteritic anterior ischemic optic neuropathy (NAION) [1]. In papilledema, the swelling is caused by axonal stasis due to elevated intracranial pressure (ICP) [2]. Conversely, in NAION, the primary cause of optic disc swelling is retinal nerve fiber layer (RNFL) edema resulting from hypoperfusion and ischemia due to disrupted vascular circulation in the optic nerve head (ONH) [3]. Additionally, certain conditions can mimic optic disc swelling, posing diagnostic and management challenges. The most common cause of pseudopapilledema is optic nerve head drusen (ONHD), characterized by bulb-like bodies within the ONH. These bodies can be buried and mimic true optic disc swelling [4]. Recommended imaging modalities for differentiating pseudo from true optic disc swelling include ONH B-scan ultrasonography, fundus autofluorescence (FAF), fluorescein angiography (FA), and optical coherence tomography (OCT) [5–9]. In patients with ONHD, ocular B-scan ultrasonography at a low gain setting can detect hyperechoic bodies in the ONH [5]. FAF may be useful for the early detection of noncalcified and buried ONHD [9]. Furthermore, FA often reveals only the staining of drusen, without any dye leakage, in contrast to conditions accompanied by actual optic disc swelling [7, 8, 10]. Drusen are typically detected as hyporeflective signal masses surrounded by hyperreflective margins in OCT images. This modality provides more information about the depth of the drusen, their morphology, and their relationship with surrounding structures. Additionally, small, deeply buried drusen can also be detected using this method [11]. Recent studies have shown that enhanced depth imaging (EDI)-OCT is an accurate and sensitive imaging modality for detecting ONHD [12]. Optical coherence tomography angiography (OCTA) is a noninvasive imaging modality that may be useful in differentiating challenging cases of optic disc edema [13–17]. Previous studies have shown that in eyes with true axonal swelling due to papilledema, some vessel density values may differ from those in eyes with pseudopapilledema [15]. Furthermore, eyes with acute NAION have been found to have lower vessel densities compared to eyes with ONHD [16]. In this study, our aim was to evaluate the OCTA features of ONHD, papilledema, NAION, and healthy eyes to assess the changes in the peripapillary microvasculature in these conditions.

## 2. Methods

### 2.1. Subjects

This prospective, comparative case-series study was conducted between December 2020 and December 2021 at the neuro-ophthalmology clinic of Rassoul Akram Hospital, Tehran, Iran. The study was approved by the Ethics Committee of the Iran University of Medical Sciences (code: IR.IUMS.REC.1399.880) and adhered to the tenets of the Declaration of Helsinki. Informed consent was obtained from all participants or their proxies.

Eyes with ONHD, papilledema, and NAION were enrolled in this study. Additionally, healthy eyes were included as the control group. Both eyes of cases with ONHD and papilledema were included in this study. For healthy subjects, one eye of each participant was randomly selected and enrolled in the study. All participants underwent a comprehensive ophthalmologic examination including measurement of best-corrected visual acuity (BCVA), checking pupillary reaction to light for detection of relative afferent pupillary defect, slit-lamp microscopy, Goldmann applanation tonometry, and dilated funduscopy. Dilated fundus photography was performed for all participants using a Topcon TRC-NW6S (Topcon America Corp., Paramus, NJ, USA) fundus camera, capturing a single-field 45° digital color retinal image (64-megapixel resolution) centered on the fovea. If the image quality was insufficient, the image was retaken.

The papilledema group consisted of patients with active and previously untreated idiopathic intracranial hypertension with lumbar cerebrospinal fluid opening pressure >25 cm H_2_O and normal magnetic resonance imaging (MRI) [2, 15, 18]. Patients with grade 4 or 5 papilledema according to the Frisén staging [19], cases with chronic papilledema (defined as a duration of diagnosis >6 months of swelling), or any signs of disc pallor were excluded. ONHD was defined as unilateral or bilateral buried or superficial drusen, verified with B-scan ultrasonography (Nidek; US-4000 Echoscan, Japan), and/or EDI-OCT (Heidelberg Engineering, Heidelberg, Germany) [4, 16, 20, 21]. In this group, cases with optic disc leakage in FA were excluded. Acute NAION was defined as acute and painless impairment of visual acuity or visual field within the last 2 weeks, along with unilateral hyperemic optic disc swelling. In this group, patients with any signs of giant-cell arteritis, including elevated erythrocyte sedimentation rate or C-reactive protein levels, were excluded. The control group consisted of subjects with a BCVA of ≥20/25, a spherical equivalent between −2.00 and + 2.00 diopters, normal optic disc appearance, and intraocular pressure ≤21 mm Hg.

In all groups, patients with ocular diseases (except for mild cataract) or a history of intraocular surgeries other than uncomplicated cataract surgery were excluded. Furthermore, OCTA images with a signal strength index of less than 40, or significant artifacts, were also excluded [22, 23]. To remove projection artifacts, the 3D projection artifact removal (PAR) algorithm, included in the software, was used to improve the quality of images [24].

### 2.2. OCT and OCTA Measurements

**Figure 1:**
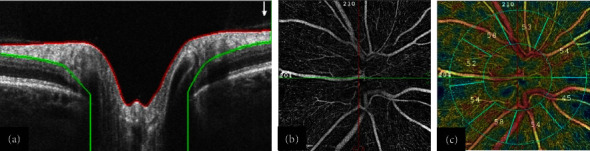
In B-scans of peripapillary optical coherence tomography (OCT), the disc boundary is characterized by Bruch's membrane opening (the termination of the retinal pigmented epithelium/Bruch's membrane), and the retinal nerve fiber layer (RNFL) is segmented from the internal limiting membrane (ILM, red line) to the RNFL posterior boundary (green line, (a)). The 4.5 × 4.5 mm^2^ optical coherence tomography angiography (OCTA) scans of the right eye are centered on the optic disc at the level of the radial peripapillary capillary (RPC) slab in a healthy subject (b). The corresponding RPC density measurements in different sectors (c). The two concentric blue circles represent 3.45- and 1.95-mm diameters. After removing large vessel signals, automatic calculations of RPC density are performed in the areas of peripapillary (between the two concentric blue circles) and the whole image in different sectors, expressed in percentages.

**Figure 2:**
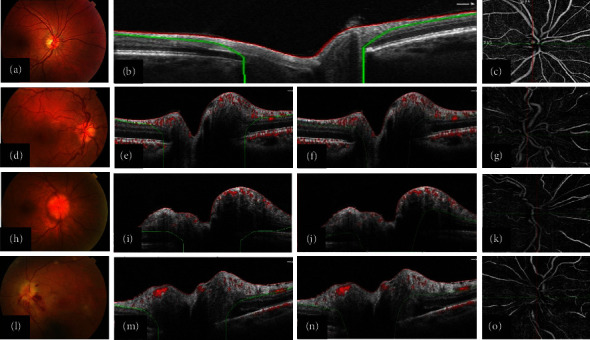
The color fundus photography, peripapillary optical coherence tomography (OCT) (before and after correcting segmentation errors), and the corresponding OCT angiography images at the level of the radial peripapillary capillary (RPC) slab in a healthy subject ((a)–(c)), an eye with optic nerve head drusen (ONHD, (d)–(g)), a case with active papilledema ((h)–(k)), and an eye with nonarteritic anterior ischemic optic neuropathy (NAION, (l)–(o)). Misidentifications of the disc boundary and retinal nerve fiber layer (RNFL) are noted in the eyes with ONHD (e), active papilledema (i), and acute NAION (m). These errors are manually corrected ((f), (j), (n)).

Peripapillary RNFL images were captured in all subjects following pupillary dilation, using the Optovue OCT (AngioVue; Optovue Inc., Fremont, CA, USA). Standard 360°, 3.4-mm-diameter circular scans were used to record average peripapillary RNFL thickness values in 4 sectors (superior, nasal, inferior, and temporal). The disc boundary was characterized by Bruch's membrane opening, and the RNFL was segmented from the inner limiting membrane (ILM) to the RNFL posterior boundary (Figure 1(a)). Any misidentification of the disc border was manually corrected by moving the position of the marginal optic disc points to their correct locations. Additionally, the segmentation lines were manually corrected in the central horizontal B-scans to measure the true thickness of the RNFL (Figure 2) [17, 24–26]. Finally, the changes were propagated to the adjacent scans using the built-in edit/propagation option. After these corrections, the RNFL thickness was recalculated and included in our analysis.

En face 4.5 × 4.5 mm OCTA images were obtained using an RTVue-XR Avanti device (software version 2017.1.0.15; AngioVue; Optovue Inc., Fremont, CA, USA) in all cases on the same day as OCT scanning. Automatic calculations of vessel densities were expressed in percentages.

In brief, after placing two concentric circles with 3.45- and 1.95-mm diameters and removing large vessel signals, capillary density was calculated. The whole image included the inside disc and peripapillary areas. The peripapillary area was between the two mentioned concentric circles, excluding the inside disc area (Figure 1(b)).

The whole image and mean peripapillary vessel densities in the segmentation of the radial peripapillary capillary (RPC) enface slab were recorded. The RPC enface image was segmented from the ILM to the RNFL posterior boundary. The software divides the peripapillary area into six parts: nasal, inferonasal, inferotemporal, superotemporal, superonasal, and temporal sectors. The software automatically removes large peripapillary vessels for better identification of microvasculature (Figure 1(c)).

Thus, the results were presented as “small vessel density” values according to Optovue machine data. The first author (K.A.) assessed each OCTA en face image for misidentification of the disc border and significant segmentation error at the RPC level. After the aforementioned manual corrections, as described previously, the vessel densities at the RPC slab were recalculated and these data were enrolled in our analysis. Detailed descriptions of OCTA image acquisition are described elsewhere [15, 16, 24, 27].

### 2.3. Statistical Analysis

IBM SPSS Statistics for Windows, Version 20.0 (Armonk, NY), was used for statistical analyses. Data were presented as mean and standard deviation (SD). Analysis of variance (ANOVA), corrected with the Tukey post hoc test, was used to compare ages, and the chi-square test was performed to compare genders between the groups. Variables reported in paired eyes, as well as BCVA, were compared using generalized estimating equations (GEEs). This method is useful for considering the correlation of paired eyes. If only one eye per patient is included in the analysis, the correlation would be replaced by zero. Therefore, GEE can be used for analyzing paired-eye data [28]. Furthermore, this method was utilized to compare OCT and OCTA parameters among groups. *P* values <0.05 were considered statistically significant.

## 3. Results

In total, 95 eyes from 73 individuals were enrolled in this study; forty-five of the cases (61.60%) were female. The mean age of the participants was 43.90 ± 17.52 years (range, 9–78). Sixteen eyes from 9 cases with bilateral ONHD, 31 eyes from 16 patients with active papilledema, and 16 eyes from 16 patients with acute NAION were enrolled. Samples of fundus photography, peripapillary OCT, and corresponding OCTA images of the optic disc at the level of RPC slab for each group are illustrated in Figure 2.

Two eyes with ONHD and 1 eye with papilledema were excluded due to the low signal strength of the captured images. Furthermore, 32 healthy eyes from 32 subjects were included as the control group. All patients were sex-matched with the controls (*P*=0.292). The control group was age-matched with the NAION group (*P*=0.094). In contrast, individuals with ONHD and papilledema were younger than the controls (all *Ps* < 0.001), but there was no difference between the ages of patients in the ONHD and papilledema groups (*P*=0.780).  Table 1 demonstrates the baseline characteristics of the participants in each group.

The best-corrected visual acuity (BCVA, in LogMAR [logarithm of the minimum angle of resolution] scale) of eyes with NAION was lower than the other groups (all *Ps* < 0.001), but there were no significant differences among ONHD, papilledema, and control groups in terms of BCVA (all *Ps* > 0.05).

### 3.1. Peripapillary RNFL Thickness

The highest mean peripapillary RNFL thickness was found in eyes with NAION (272.23 ± 115.63 *µ*m), followed by papilledema (235.92 ± 85.06 *µ*m), ONHD (224.39 ± 82.56 *µ*m), and healthy eyes (103.31 ± 9.36 *µ*m), respectively.

The mean and sectoral peripapillary RNFL thicknesses were significantly higher in all patient groups than in healthy subjects (all *Ps* ≤ 0.001), except for the temporal sector, which was not statistically significant between the healthy (73.86 ± 12.00 *µ*m) and the ONHD (130.50 ± 75.30 *µ*m) groups (*P*=0.419).

There was no significant difference regarding this parameter among eyes with ONHD, papilledema, and NAION (all *Ps* ≥ 0.05, Table 2).

### 3.2. Peripapillary Vessel Density

The vessel densities were measured after excluding the large vessels via the device's software to calculate only the capillary densities. For the mean peripapillary vessel density values, the highest values belonged to the ONHD (52.46 ± 2.36%), control (52.06 ± 2.07%), papilledema (48.90 ± 4.19%), and NAION (46.43 ± 6.01%) groups, respectively. The mean peripapillary vessel density did not differ between the ONHD and control groups (*P*=1.000), nor between the NAION and papilledema groups (*P*=0.216). Nevertheless, this value in the ONHD and control groups was significantly higher than that in the NAION and papilledema groups (all *Ps* < 0.05).

In the sectors of the peripapillary area, the vessel density values in just the nasal sector were not statistically significant between any groups (all *Ps* > 0.05). For the other sectors, the details are mentioned in Table 3. For example, in comparison to the controls, there were significant vessel density reductions in eyes with papilledema at the temporal (54.85 ± 2.32 vs 49.64 ± 7.47%) and superotemporal (54.85 ± 3.03 vs 49.03 ± 9.16%) sectors (*P*=0.001 and *P*=0.009, respectively).

In eyes with NAION, the vessel densities were significantly lower than those in healthy eyes in all sectors except the nasal and inferonasal sectors (*P*=0.358 and *P*=0.057, respectively). Interestingly, no significant differences in vessel densities were observed in any sectors between the papilledema and NAION groups (all *Ps* > 0.05).

## 4. Discussion

The use of OCTA has become increasingly prevalent in noninvasive imaging for assessing the ONH. By detecting temporal changes in blood flow within vessels across different ONH and peripapillary segments, OCTA provides valuable insights [13]. Notably, even in cases with edematous or elevated optic discs, where peripapillary OCTA image quality may vary, the overall accuracy of OCTA remains acceptable [17]. Despite its capability to detect alterations in vessel densities in eyes with swollen discs, discrepancies persist among OCTA findings in swollen discs of different etiologies [14–16, 29]. Moreover, this technique is more prone to artifacts compared to FA imaging [30]. Therefore, FA remains a routine diagnostic tool for the assessment of swollen discs, despite being an invasive and time-consuming method compared to OCTA scanning. FA often reveals staining without evidence of leakage in cases of ONHD, distinguishing them from papilledema or NAION, which typically exhibit signs of dye leakage [31]. In conditions with true or pseudo swollen discs, alterations in blood flow, due to various causes, can be detected as a delay in perfusion or hypoperfusion in the early stages of the disease [2, 3, 29, 32, 33]. However, dye leakage may obscure the vessels of the optic disc, making it difficult to properly evaluate the ONH vasculature in eyes with true disc swelling [29]. On the other hand, if the blood flow speed is below the slowest detectable flow, the OCTA device will be unable to detect the motion contrast between two successive B-scans, which can underestimate vessel densities. In cases with ONHD, papilledema, and NAION, low blood flow speed in the ONH may hypothetically alter the quality of OCTA scans. However, this problem is resolved in new generations of OCTA devices by using a higher speed system and selecting different time frames between the B-scans to increase image quality [30].

In this study, we assessed the OCTA measurements of the ONH and the peripapillary area in healthy subjects, as well as in eyes with ONHD, papilledema, and NAION. Furthermore, we measured the mean peripapillary RNFL thickness among the study groups. We detected a decrease in peripapillary vessel density values in eyes with papilledema and NAION when compared to the controls. However, this reduction was not observed in the ONHD group.

As it is known, peripapillary RNFL thickness increases in eyes with true optic disc swelling, including papilledema and acute NAION [34]. In ONHD, the adjacent RNFL thickness may decrease focally in the presence of superficial drusen, while buried drusen may lead to an increase in RNFL thickness [4, 35–37]. Our study revealed an increase in peripapillary RNFL thickness in the ONHD group, similar to the NAION and papilledema groups, although statistical significance was not reached. Differentiating true from pseudodisc swelling based solely on RNFL thickness proved to be challenging [6, 15, 38, 39]. This discrepancy can be explained by the etiology of pseudopapilledema and the location of ONHD.

Previous studies have reported varied and sometimes paradoxical findings in OCTA scans of eyes with ONHD, influenced by factors such as drusen size, location, and depth [15, 16, 40, 41]. Abri Aghdam et al. found a decreased RPC vessel density in the peripapillary area of eyes with ONHD compared to healthy eyes [16]. Fard et al. reported that in eyes with pseudopapilledema (including eyes without ONHD), RPC vessel density decreased in the peripapillary retina compared to healthy controls [15]. These alterations in vessel density in eyes with ONHD can be explained by the focal compressive effect of drusen on retinal blood flow [41].

Axonal stasis in papilledema and ischemic conditions in NAION contribute to decreased vessel density values in the ONH and peripapillary retina. Our study confirmed a decrease in mean peripapillary vessel density in NAION and papilledema compared to healthy subjects. Abri Aghdam et al. reported the same finding in eyes with NAION; all peripapillary vessel density values demonstrated a reduction [16]. Hata et al. showed a decrease in all vessel density values of the peripapillary area in eyes with NAION compared to healthy eyes [42]. These alterations in vessel densities can be explained by the ischemic condition in eyes with NAION [43]. In the papilledema group, we observed a lower mean peripapillary vessel density compared to the controls. Despite the nonischemic condition in papilledema, artifacts from axonal stasis and edema can affect the true detection of capillary structures by OCTA, leading to incorrect measurements. Furthermore, the presence of large peripapillary vessels could exacerbate the artifact effect [2, 15, 44]. To counteract the artifact of large peripapillary vessels, Fard et al. designed a custom image analysis software to remove the contribution of these vessels in inducing artifacts during the measurement of the density of the small vessels in swollen discs. After the removal of large vessels, no significant differences in peripapillary vasculature values were observed between the papilledema and healthy control groups [15]. This was in contrast to findings with the commercial software of their device (Optovue OCT [software version 2016.1.0.90], AngioVue; Optovue, Inc., Fremont, CA, USA), which showed lower peripapillary vasculature values in the papilledema group. In our study, a significant decrease in mean peripapillary vessel density was observed in the papilledema group compared to the ONHD group. Other differences in RPC vessel density values were not statistically significant. Yalcinkaya Cakir et al. demonstrated a significant decrease in the peripapillary density of the deep capillary plexus in eyes with idiopathic intracranial hypertension (IIH) compared to the ONHD group [45]. Fard et al. found no differences in vessel density values between eyes with papilledema and pseudopapilledema based on the outputs of the commercial software of their OCTA device. After removing large peripapillary vessels, a significant decrease in the whole image and nasal sector peripapillary capillary density was observed in eyes with pseudopapilledema (including eyes with and without ONHD) compared to the papilledema group [15]. This discrepancy between our results and their findings could be explained by differences in the location and depth of drusen, the stage of papilledema, different software protocols used to mask large vessels, and the unpredictable effects of potential OCTA artifacts. Although the software used in our study could automatically mask large peripapillary vessels, remaining artifacts may cause alterations in vessel density measurements. We observed a nonsignificant decrease in the whole image and mean peripapillary vessel densities in eyes with NAION compared to the papilledema group. Rougier et al. demonstrated that eyes with NAION had a less visible peripapillary RPC network, in contrast to eyes with papilledema [29]. This finding can be explained by the ischemic feature of NAION that involves the ONH vasculature. On the other hand, there is only axonal stasis in the early stages of papilledema, and a peripapillary ischemic condition is not a common finding [14, 15, 29, 43, 46].

Although some parameters have been suggested in our study and previous publications to differentiate true from pseudo-optic disc swelling using OCTA, they are not reliable and reproducible enough to establish diagnostic criteria. This limitation can be explained by the mechanism of OCTA imaging, which is based on the detection of blood flow, regardless of the etiology. Any disturbances in blood flow due to vessel wall compression by ONHD, blood stasis due to raised ICP, and changes in the caliber or structure of vessels in inflammatory conditions may lead to alterations in blood flow detection and vessel density measurements with OCTA [32]. Moreover, these etiologies may present together. For example, if ONHD is large enough, it can disrupt the blood flow to the ONH and lead to blood stasis and ischemia. Furthermore, patients with ONHD are at an increased risk of progression to NAION compared to healthy subjects [47–49].

This study had some limitations. First, a relatively small number of subjects were included, with sample heterogeneities, including different stages of active papilledema. Second, the NAION and control groups were not age-matched with the ONHD and papilledema groups. However, NAION typically occurs in elderly patients, and the age factor had no significant effect on the ONH microvasculature [27, 50]. Third, we only evaluated the RPC segment, and other slabs were not assessed. However, this vascular bed plays the most crucial role in maintaining the normal function of the RNFL [50]. Furthermore, due to their parallel structures with low anastomoses, RPCs are potentially susceptible to ischemic or compressive injuries [51, 52]. Fourth, different types of artifacts, especially segmentation and shadow artifacts due to ONH swelling, might influence the measurements [15, 44]. To minimize the effect of these artifacts on our results, we manually corrected disc boundary misidentification and segmentation errors, as described in detail previously. Fard et al. designed a custom image analysis software to eliminate these errors and artifacts in the measurement of small vessel density in eyes with swollen discs [15]. However, Anvari et al. demonstrated that although the accuracy of OCT and OCTA images in swollen discs with NAION is decreased, the RNFL thickness and the RPC density did not change statistically significantly after corrections for the errors [17]. Fifth, we did not use macular ganglion cell complex (GCC) imaging as an indicator of early neuronal loss. It is known that GCC analysis is a potential indicator of neuronal loss in the follow-up of eyes with papilledema to detect signs of inner retina layers' atrophy in the presence of remnant RNFL edema, especially in the early stage of the disease [53]. However, the role of macula GCC in the follow-up of NAION and ONHD is not clearly stated. Therefore, we did not include macula GCC for comparison among multiple groups as it was not the focus of our study.

In conclusion, peripapillary vascular density is affected during the course of ONHD, papilledema, and NAION. Manual corrections of misidentification of disc boundaries and segmentation errors of peripapillary vascular layers may reduce artifacts induced by edema during OCTA imaging in eyes with acute optic disc swelling. With these corrections, OCTA imaging can be considered a precise modality for quantifying vessel densities in such cases. However, further studies are needed to evaluate the application of OCTA for differentiating true from pseudo-optic disc edema.

## Figures and Tables

**Table 1:** Baseline characteristics of the participants in each study group.

Variable	Healthy (control, 32 eyes of 32 subjects)	ONHD (16 eyes of 9 subjects)	Papilledema (31 eyes of 16 subjects)	NAION (16 eyes of 16 subjects)	*P*
Age (years, mean ± SD)	47.75 ± 14.50	25.67 ± 10.51	32.19 ± 15.30	58.19 ± 12.28	<0.001
Female, *n* (%)	21 (65.6)	5 (55.6)	12 (75)	7 (43.8)	0.292
BCVA (LogMAR, mean ± SD)	0.00 ± 0.00	0.05 ± 0.04	0.03 ± 0.04	0.74 ± 0.64	<0.001

SD = standard deviation; ONHD = optic nerve head drusen; NAION = nonarteritic anterior ischemic optic neuropathy. Statistical significance was tested by analysis of variance (ANOVA), corrected with Tukey post hoc test (for age), chi-square test (for gender), and generalized estimating equations (GEE for BCVA).

**Table 2:** Peripapillary RNFL thickness measurements in healthy subjects, ONHD, papilledema, and NAION.

Region	Healthy eyes, *n* = 32	ONHD, *n* = 16	Papilledema, *n* = 31	NAION, *n* = 16	*P*
Average RNFL (*µ*m)	103.31 ± 9.36	224.39 ± 82.56	235.92 ± 85.06	272.23 ± 115.63	<0.001^∗,⁑,⁂^
Superior RNFL (*µ*m)	88.71 ± 10.85	259.44 ± 104.57	253.04 ± 137.73	320.63 ± 140.40	<0.001^∗,⁑,⁂^
Nasal RNFL (*µ*m)	103.81 ± 12.72	223.94 ± 93.55	263.17 ± 104.64	241.94 ± 86.89	<0.001^∗,⁑,⁂^
Inferior RNFL (*µ*m)	146.84 ± 15.59	283.69 ± 109.16	241.69 ± 101.25	312.88 ± 138.96	<0.001^∗,⁂^, 0.001^⁑^
Temporal RNFL (*µ*m)	73.86 ± 12.00	130.50 ± 75.30	188.63 ± 106.27	213.50 ± 182.27	<0.001^⁑,⁂^

RNFL = retinal nerve fiber layer, ONHD = optic nerve head drusen; NAION = nonarteritic anterior ischemic optic neuropathy. Statistical significance tested by generalized estimating equations (GEE). ^∗^Significant difference between mean values of ONHD and healthy eyes. ^⁑^Significant difference between mean values of papilledema and healthy eyes. ^⁂^Significant difference between mean values of acute NAION and healthy eyes.

**Table 3:** RPC vessel density measurements in healthy subjects, ONHD, papilledema, and NAION.

Region	Healthy eyes, *n* = 32	ONHD, *n* = 16	Papilledema, *n* = 31	NAION, *n* = 16	*P*
Whole image (%)	49.62 ± 1.81	49.74 ± 2.16	47.25 ± 4.57	44.98 ± 4.98	<0.001^⁂^, 0.002^╪^
Mean peripapillary (%)	52.06 ± 2.07	52.46 ± 2.36	48.90 ± 4.19	46.43 ± 6.01	<0.001^⁂,^^╪^, 0.008^⁑^, 0.018^┼^
Nasal (%)	49.09 ± 2.84	49.96 ± 2.49	48.93 ± 6.02	46.34 ± 6.26	All *Ps* > 0.05
Inferonasal (%)	50.26 ± 3.64	52.56 ± 4.03	47.36 ± 7.11	45.70 ± 7.03	0.005^╪^, 0.023^┼^
Inferotemporal (%)	55.30 ± 4.32	57.30 ± 4.30	52.07 ± 7.59	48.88 ± 10.19	0.004^╪^, 0.015^⁂^
Superotemporal (%)	54.85 ± 3.03	54.47 ± 4.33	49.03 ± 9.16	45.05 ± 9.77	<0.001^⁂^, 0.002^╪^, 0.009^⁑^
Superonasal (%)	49.60 ± 3.80	48.93 ± 7.27	46.22 ± 7.03	42.44 ± 8.30	0.003^⁂^, 0.032^╪^
Temporal (%)	54.85 ± 2.32	54.61 ± 3.31	49.64 ± 7.47	48.28 ± 5.00	<0.001^⁂^, 0.001^⁑^, 0.004^╪^, 0.013^┼^

RPC = radial peripapillary capillary, ONHD = optic nerve head drusen; NAION = nonarteritic anterior ischemic optic neuropathy. Statistical significance tested by generalized estimating equations (GEE). ^∗^Significant difference between mean values of ONHD and healthy eyes. ^⁑^Significant difference between mean values of papilledema and healthy eyes. ^⁂^Significant difference between mean values of acute NAION and healthy eyes. ^┼^Significant difference between mean values of papilledema and ONHD. ^╪^Significant difference between mean values of acute NAION and ONHD.

## Data Availability

The datasets used during the current study are available at the Eye Research Center, The Five Senses Health Institute, Iran University of Medical Sciences, Tehran, Iran. The data are not available publicly due to confidentiality. However, upon a reasonable request, the data can be obtained from the corresponding author.
